# Gender and occupational differences in post-terrorist mental health outcomes among emergency responders with and without crisis intervention

**DOI:** 10.1093/eurpub/ckac129.338

**Published:** 2022-10-25

**Authors:** U Wesemann, M Mahnke, S Polk, A Bühler, G Willmund

**Affiliations:** 1 Department of Psychiatry, Psychotherapy, Bundeswehr Hospital, Berlin, Germany; 2 Fire Department Berlin, Berlin, Germany; 3 Max Planck Institute for Human Development, Berlin, Germany

## Abstract

**Introduction:**

After the Berlin terrorist attack at Breitscheidplatz, the gender-specific evaluation of emergency responders (ER) revealed higher perceived levels of paranoid ideation among females. The occupation-specific evaluation revealed higher hostility among police officers and a lower quality of life among firefighters. The aim of this study was to examine the outcome of crisis intervention (CI) provided for ER deployed to this terrorist attack.

**Methods:**

In total, N = 55 ER were included (n = 37 with and n = 18 w/o CI). Stress, quality of life, post-traumatic stress disorder, and current psychological stress were assessed 4 months after the attack.

**Results:**

ER with and w/o CI were compared. Participants with CI showed lower quality of life in psychological health (t(53)=2.01, p=.050) and higher depressive symptomatology (t(44) = 2.51, p=.016). Females with CI showed lower quality of life in social relationships (t(12)=2.46, p=.030), whereas males showed more posttraumatic stress symptoms (t(39)= .32, p=.026). Emergency responders from NGOs with CI had higher phobic anxiety (t(9.2)=2.72, p=.023). Emergency medical technicians with CI showed more somatic (t(7.5)=2.52, p=.037) and depressive (t(8)=2.30, p=.050) symptoms.

**Conclusions:**

This study provides evidence for differences in the mental health burden for ER with and w/o CI, in general and for subgroups of gender and occupation. There is no conclusive explanation for why ER with CI score worse on certain measures. It is possible that CI had a harmful influence due to the reinforcement of negative emotions in some parts of measures like the Critical Incident Stress Debriefing (CISD). While it is known that ERs are vulnerable to develop mental problems, and appropriate and timely help is recommended, it is important to critical evaluate the methods used and to take also into account the identified gender and occupational differences. Further research is needed to better understand the interaction of risk factors.

